# Built Environment and Assistive Technology Design in Residential Aged Care: A Scoping Review and Mapping of Evaluation Methods and Measures to the World Health Organization’s International Classification of Functioning, Disability and Health

**DOI:** 10.3390/ijerph23070869

**Published:** 2026-07-03

**Authors:** Libby Callaway, Natasha Layton, Phillippa Carnemolla, Lisa Licciardi, Maryam Gusheh, Em Bould

**Affiliations:** 1 Rehabilitation, Ageing and Independent Living (RAIL) Research Centre, Faculty of Medicine, Nursing and Health Sciences, Monash University, Frankston 3199, Australia; natasha.layton@monash.edu (N.L.); lisa.licciardi@monash.edu (L.L.); em.bould@monash.edu (E.B.); 2 Department of Occupational Therapy, Faculty of Medicine, Nursing and Health Sciences, Monash University, Frankston 3199, Australia; 3 National Centre for Healthy Ageing, Faculty of Medicine, Nursing and Health Sciences, Monash University, Frankston 3199, Australia; maryam.gusheh@monash.edu; 4 School of Built Environment, Faculty of Design, Architecture and Building, University of Technology Sydney, Ultimo 2007, Australia; phillippa.carnemolla@uts.edu.au; 5 School of Architecture, Faculty of Arts, Design and Architecture, Monash University, Caulfield 3162, Australia

**Keywords:** aged care, nursing homes, environmental design, assistive products, methodology, measurement

## Abstract

**Highlights:**

**Public health relevance—How does this work relate to a public health issue?**

- The World Health Organization (WHO) recognizes the global growth in ageing populations, with every country experiencing an increasing size and proportion of older persons, and forecasts indicating that the number of people aged over 80 globally will triple between 2020 and 2050.
- Whilst there are common health conditions associated with ageing, variations in the health of older adults can also be due to the physical and social environments they experience.

**Public health significance—Why is this work of significance to public health?**

- Older adults often require supported living environments, including residential aged care (RAC), due to age-related health conditions, and it is important to consider both features of the built environment and assistive technology design that may act as a facilitator or barrier to health outcomes.
- To date, there has been a limited coordinated effort to identify methods and measures used internationally to evaluate RAC-built environment and assistive technology design, and this review addresses that gap. Mapping methods and measures to the WHO ICF framework (which offers use of internationally recognized terminology) offers significant additional value in assisting aged care researchers, providers and funders to consider holistic approaches to the design, delivery and evaluation of RAC-built and technology environments, and their impact on health outcomes of older adults.

**Public health implications—What are the key implications or messages for practitioners, policy makers and/or researchers in public health?**

- Built environment and assistive technology can be a facilitator or barrier to outcomes of older adults living in RAC; however, research identified through this study has primarily been undertaken in high-income countries with mixed populations; most often examined built environment and assistive technology separately, rather than as an ecosystem of environmental interventions; and—of the studies identified—25% did not collect data from resident populations, thereby missing perspectives of the primary users of the support environments being evaluated.
- Methods and methodologies used to evaluate environmental interventions vary significantly, further limiting the generalizability of the research that exists.

**Abstract:**

Globally, the growth of ageing populations is significant, with more people requiring supported living environments, including residential aged care (RAC). Given the influence of the environment on health outcomes, it is important to consider approaches to evaluate aged care design, including both the built environment and products and technology. With the overarching aim to identify the scope of RAC-built environment and assistive technology design interventions and the way this data is captured methodologically, this review (i) identified methods and measures used to evaluate RAC-built environment and assistive technology design, and examined populations these methods and measures were used with, and (ii) mapped identified approaches to the International Classification of Functioning, Disability and Health (ICF). An *a priori* review protocol was developed, and a scoping review was then conducted. Eight databases were searched for publications between January 2000 and February 2023, resulting in 81 included studies, which were then mapped to ICF activity, participation and environment domains. Twenty methods and 16 methodologies were identified. Sixty-one articles collected data directly from resident populations, primarily including older adults (n = 52). Forty-nine publications reported on the evaluation of built design, 23 reported on products and technology, and nine reported on both, but with limited inclusion of valued participation as a goal or outcome. While some participatory methods were identified, 25% of the studies did not include consumer perspectives. Analyzing aged care design can identify ways to facilitate, or remove barriers to, healthier spaces and lives in RAC. Use of internationally recognized terminology and an integrative lens on the relationship between technology and environmental design is recommended.

## 1. Introduction

Built environment refers to the human-made surroundings where people live, work and play [1]. Internationally, the important role that the built environment plays, and its impact on the functioning, dignity and quality of life of older adults, is well recognized [2,3]. With the growing recognition of the benefits of achieving healthier spaces and lives for older adults, emphasis has been placed on the consideration of human rights, participatory approaches and cultural contexts in environmental design [4,5]. Assistive technology—defined by the World Health Organization (2020) [6] as an umbrella term for assistive products and the services needed to select, provide, fit, and train people on their use—has also been recognized to maintain or improve functioning, and facilitate health outcomes in older adulthood [7,8,9].

The World Health Organization (WHO) recognizes the global growth in ageing populations, with every country internationally experiencing an increasing size and proportion of older adults, and forecasts indicating that the number of people aged over 80 years globally will triple between 2020 and 2050 [10,11]. Internationally, the majority of care for older adults is provided to people in their homes; however, in many countries, the greatest proportion of government aged care spending is on residential aged care (RAC) [12,13,14]. Built design and modification, and assistive technology, are key environmental interventions that can support both equity and personally meaningful outcomes (including participation and quality of life) in older adulthood [4,15]. Given the interaction of assistive technology and environments, it is essential for designers, providers, and funders to consider and address the needs of older adults [5].

As RAC environments have been evolving in recent decades and with a growing global trend towards the concept of aging-in-place, RAC now needs to meet multiple and complex needs of residents [13,16]. The importance of the physical environment and environmental design for older adult health and well-being is well recognized [17,18]. Internationally, some countries have developed regulatory instrumentation or rights-based recommendations regarding the provision of long-term care that offers residents physical and attitudinal environments that are dignified, safe and respectful [19,20,21]. As an example of a targeted policy approach, a recent Royal Commission into Aged Care Quality and Safety in Australia identified the role of RAC environmental design to be either “supportive, familiar and therapeutic, or a barrier to independent functioning and high quality of life” [22] (p. 105). A range of recommendations stemmed from this Royal Commission, including that the government should undertake work to guide residential aged care design by developing and publishing a set of national aged care design principles and guidelines on accessible and dementia-friendly design, innovation, and safety features in aged care [23]. Acting on this recommendation, the Australian government department of health, disability and ageing has completed consumer, community and aged care sector consultations; developed a discussion document on assistive technologies and home modifications scheme for in-home aged care [24]; drafted a new residential aged care accommodation framework [25]; and delivered a final report on the development of national aged care design principles and guidelines released in September 2023 [26].

Despite these efforts internationally, a recent Cochrane review highlighted that there is currently insufficient research evidence to draw conclusions about the impact of environmental design changes for older adults living in RAC. The authors proposed that this was because outcomes directly associated with the design of the built environment in a supported setting are difficult to isolate from other influences, such as resident health changes, adjustments in care practices over time, or variations in staff providing support across shifts [17]. The review highlighted that aged care design requires rigorous evaluation, using efficient and effective methods that can elicit information on the intersection of the built environment, assistive technology, and support delivery. This is because built design alone is unlikely to result in improved outcomes for residents or the operation of RAC—rather, built design should be considered in the context of the preferences and needs of the residents, the perspectives of staff at the aged care facility, and the model of care provided [27,28]. Adding to this, members of the environmental design special interest group (part of Dementia Alliance International, which is an international advocacy and support group led by people living with dementia) have recently called for investment in participatory research focused on assistive technology and environmental design that is undertaken with both people living with dementia and cross-disciplinary teams working with them [5]. Evaluation approaches that are responsive and appropriate to the range of resident populations and complex, dynamic aged care workspace environments have also been called for to better inform design [29,30].

While a range of environmental design and assistive technology theories and frameworks exist, the World Health Organization’s International Classification of Functioning, Disability and Health (ICF) was selected as the framework of choice due to its international adoption and the standardized terminology it offers. The ICF is an international framework used to describe and classify information related to health, disability and functioning, and is underpinned by the concept that a person’s level of functioning and disability is the result of interactions between health condition/s, personal factors and environmental factors [31]. The ICF explicitly positions ‘products and technology’ within environmental factors, thereby contextualizing assistive technology alongside other aspects of environmental and spatial design. The use of the ICF framework within scoping reviews has been encouraged, as the framework offers the opportunity to use consistent language understood internationally [32]. Specific to older adults and environmental design, the ICF has also proven useful in reviews to facilitate the grouping of environmental factors that may impact health outcomes, and which may otherwise be difficult to categorize [33,34].

The growing international demand, together with significant government reforms underway and emerging guidance on environmental design, creates a complex context for evaluation. Accordingly, this study adopted a two-stage approach with the aim to examine and categorize factors related to the RAC-built environment and assistive technology design together within the overarching conceptual framework of ICF. The first stage was to undertake a scoping review of the literature, with two research posed questions: *what methods and measures have been used to evaluate RAC-built environment and assistive technology design internationally*; and *which populations were these methods and measures used with?* The second stage of work was then implemented to address the following research question: *how do the identified methods and measures map to the WHO ICF activity, participation and environment domains and subdomains?*

## 2. Materials and Methods

### 2.1. Scoping Review Method

In the first stage of work, Arksey and O’Malley’s [35] and Levac et al.’s [36] methodology for completing scoping reviews was used to develop a structured *a priori* protocol (available upon request), which was then methodically applied to guide the search for peer-reviewed and grey literature to address research aims 1 and 2. The scoping work included five key phases: (i) identifying the research aims and objectives; (ii) identifying relevant studies; (iii) study selection; (iv) charting the data; and (v) collating, summarizing, and reporting the results. Review reporting adhered to the Preferred Reporting Items for Systematic Reviews and Meta-Analyses-extension for Scoping Reviews (PRISMA-ScR) [37]. Scoping review methodology was selected for this review because the approach allows researchers to examine the existing literature on emerging topics of research, providing an overview and highlighting gaps that exist—consistent with this approach, the risk of bias or methodological limitations of the included studies was not assessed [37].

### 2.2. Inclusion Criteria

Inclusion criteria were that articles were published in full text in English, between the years of January 2000 and the completion of the database search in February 2023, and inclusive of populations of adults aged 18 years or over (or mixed populations with at least a 50% sub population that includes adults). Studies reporting on methods and measures used to evaluate built and/or technology-enabled design in RAC (including quantitative, qualitative, and mixed methods designs) were included. In addition, studies that report standardized measurement instruments (defined as measurement instruments with clear procedures for administration and scoring) that were used—or which focused on the development or psychometric evaluation of measurement instruments or their cultural/linguistic adaptation/translation—in the evaluation of RAC-built and/or technology-enabled design were also included. Systematic or scoping reviews, and studies of disability-specific settings that were not described as or included as a component of RAC were excluded.

### 2.3. Information Sources and Search Strategy

With the input from two expert university librarians, one in health and one in design, search terms were established for each of the key concepts (see Table 1). The combination of keywords, Boolean operators and truncations was varied in accordance with the indexing of each database searched: Ovid MEDLINE(R); EmCARE; CINAHL Plus; CABI (Global Health); Scopus; Avery Index to Architectural Periodicals (ProQuest); Australian Architecture database (Informit); Art & architecture source (EBSCOhost); and Compendex (Elsevier).

### 2.4. Study Selection

One author (E.B.) with input from the two expert university librarians completed the search on 28 February 2023. The resultant records were initially imported into the referencing software Endnote (EndNote X8, Clarivate Analytics, Philadelphia, PA, USA), and after removing duplicates, the 1222 articles were exported into an online abstraction tool (Covidence Software (https://www.covidence.org/ accessed on 31 March 2023)) to support the screening process, which happened in two stages: title and abstract screening and then full-text screening. All articles were screened against the inclusion and exclusion criteria by two independent reviewers (E.B. and either D.R. or L.C.). Discrepancies were resolved through a third reviewer (L.C. or P.C.) screening any conflicted articles, with a consensus audit trail recorded in a spreadsheet developed in Microsoft Excel (Microsoft Corporation, Redmond, WA, USA).

### 2.5. Data Extraction and Analysis

Data extraction linked to study aims 1 and 2 was completed by two authors (L.C. and E.B.) using a spreadsheet developed in Microsoft Excel. The spreadsheet captured the following information: title of article, authors, year of publication, country of data collection, study design, sample size, study duration, location and size of aged care setting, target population, and the methods and measures used. Anticipating significant variations in study designs and research methods used, analyses were undertaken using descriptive statistics (calculated in Microsoft Excel) and a qualitative narrative synthesis approach, rather than a specific meta-analysis.

In the second stage of work, the ICF was used to address study aim 3. Data extraction in this stage of work was completed by authors with experience in the ICF (N.L., P.C. and L.L.)—and then reviewed by the lead author (L.C.)—using a spreadsheet developed in Microsoft Excel. The spreadsheet captured the following information: environmental factors (around the person), including subcategories of built environment (indoor), built environment (outdoor), natural environment, support and relationships and attitudes, and services, systems and policies; and activities and participation (related to the person), including subcategories of self-care, interpersonal interactions and relationships, communication, learning and applying knowledge, domestic life, community, social and civic life, mobility and major life areas (education, work). This second stage of work facilitated a detailed analysis of the components of each included publication related to the environment, activities and participation as defined in the ICF [31]. This approach has been used similarly by other authors previously [32,33] and provides an integrative analysis of all environmental factors, including the role of assistive technology and built design, which are otherwise often viewed as discrete and thus their interrelationships could be missed.

## 3. Results

Results are presented to firstly address study aim 1 (identifying methods and measures used to evaluate RAC-built environment and assistive technology design internationally) and study aim 2 (examine the populations, methods and measures with whom they are used). Results addressing study aim 3 (mapping the identified methods and measures to the WHO ICF activity, participation and environment domains and subdomains) are then presented.

**Figure 1:**
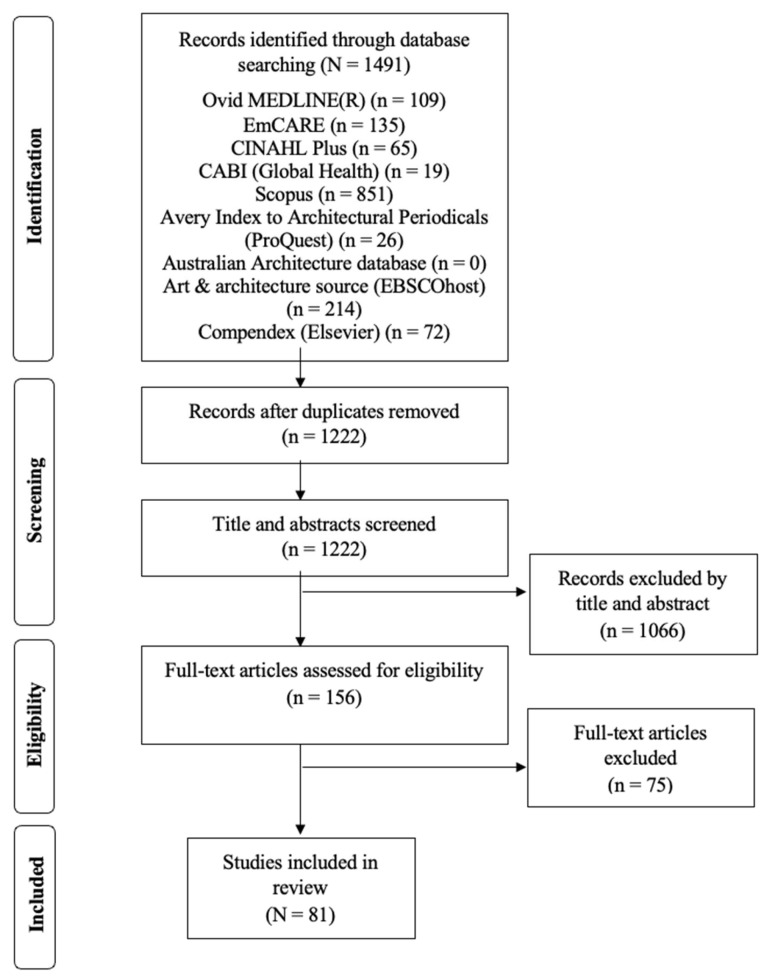
PRISMA-ScR flow chart.

Figure 1 presents the scoping review results, reported using the PRISMA-ScR flow chart [37]. The Preferred Reporting Items for Systematic reviews and Meta-Analyses extension for Scoping Reviews (PRISMA-ScR) Checklist is provided in Table S1. Table S2 lists all 81 articles that met eligibility for inclusion in the review, including the year of publication, author/s, publication title and Digital Object Identifier (DOI) (see Table S2).

### 3.1. Study Characteristics

Included articles were published between 2004 and February 2023, with the majority (n = 50, 62%) published over the past decade (i.e., from 2017 onwards). Studies were carried out in RACs across 28 countries, the highest in Australia (n = 18), followed by the United States of America (USA) (n = 7), China (n = 7), the United Kingdom (UK) (n = 5), Japan, Sweden, and The Netherlands (n = 4 in each of these countries). Four included studies reported on RACs across multiple countries: Asia, Australia, Europe and the USA (n = 1), Australia and Korea (n = 1), Romania and Cyprus (n = 1), and the UK, USA and Scandinavia (n = 1). The sample size and proportion of the examined population in the included studies varied, from as few as eight participants from one RAC to a sample of aged care residents across 7785 aged care settings. Only two studies included indigenous populations [38,39].

### 3.2. Methods and Measures Used to Evaluate Built Environment and Assistive Technology Design in RAC

Sixteen articles were qualitative, 44 quantitative and 21 used mixed methods, with included publications incorporating a total of 20 research methods (see Table 2). Questionnaires/surveys, observations, and interviews were most commonly used, but the number of methods within each publication varied. Twenty-three articles (28%) utilized one method, 27 (33%) two methods, 18 (22%) three methods, six (7%) four methods, six (7%) five methods, and one (1%) six methods.

Eight articles described the development of a measure focused on the built environment and/or assistive technology design in RACs (see Table 3a). Across the remaining 73 articles, a total of 34 published measures were used. Of these, seven measures had clearly demonstrated a focus on the built environment and/or assistive technology design in RACs (see Table 3b).

### 3.3. Populations the Methods and Measures Have Been Used with

Table 4 reports on which populations each of the methods had been used with. A total of 61 articles collected data directly from resident populations, which primarily included older adults (n = 52). Presented in Table 5 are the populations each of the 15 measures had been used with, which shows that 11 of the measures were used with multiple populations.

### 3.4. Domains of the WHO ICF Framework

Table S3 reports on the second stage of work, where each of the 81 included publications was examined, with data extracted and mapped to the ICF environment, activities, and participation domains (and subdomains). Forty-nine publications reported on the evaluation of built design, 23 reported on products and technology, and nine reported on both. Specific to activities and participation, community, social and civic life (n = 41 articles), interpersonal interactions and relationships (n = 40) and domestic life (n = 36 articles) were most commonly examined, whilst ‘major life areas (education, work)’ (n = 5 articles) and ‘learning and applying knowledge’ (n = 3 articles) were least examined (see Table S3).

## 4. Discussion

This review has scoped methods and measures used to evaluate the built environment and assistive technology design in residential aged care, highlighting a range of evidence gaps and imperatives for future research.

Globally, people are ageing, and the demand for supported living environments, including residential aged care (RAC), is growing. For these reasons, understanding approaches to evaluate design interventions and their impact on outcomes is important. Evaluation of the built environment and assistive technology design in RAC, including ways these environmental interventions can enhance or pose barriers to health and well-being, is crucial. This scoping review offers a novel contribution examining methods and measures used to evaluate built environment and assistive technology design in RAC. The additional phase of work, mapping methods and measures identified for the ICF, offers the availability of internationally recognized terminology for and insights into domains that have been examined in the evaluation of RAC-built environment and assistive technology design.

Given the large scale and growing government investment in aged care services internationally, the evaluation of an RAC design that can facilitate or pose barriers to healthy spaces and healthy lives for residents—including approaches that analyze the built environment on human health—is a current imperative [26]. Upgrading or modification of the existing environment to better meet the needs of older adults, including those with mild cognitive impairment or dementia, is also encouraged [18]. However, this current review identified only a limited body of research describing the methods and measures used to evaluate such environments. Of the studies identified, they most often looked at either built design or assistive technology, rather than the interaction of the two as an ecosystem of environmental interventions that build capability [15].

The ICF has previously been identified to provide a comprehensive analysis of experiences and needs from the person’s perspective, including older adults [32,34]. Mismatch in the older adult and aged care environments can lead to poor outcomes; to fix this, interventions need to enhance alignment between the physical and social environment and personal agency and dignity [63,64]. However, the current research has demonstrated that—specific to methods and measures used to evaluate RAC design—some subdomains of the environment, activities and participation were less often in focus as part of RAC evaluation, yet could impact outcomes achieved. Following, only nine articles retrieved examined both the built and assistive technology environment as an ecosystem, whilst the majority examined one or the other in isolation. Given the identified importance of both built and technology design (and their interaction) to influence activities and participation, the existing literature risks being limited in its evaluation scope. This finding aligns with recent reviews applying ICF concepts, which found that environmental interventions are often under-reported and that all domains of the ICF should be considered when developing care models and support services based on the needs of older adults [9,33].

Whilst 28 countries were represented in the evidence identified in the current review, almost two-thirds (60%) of the studies were undertaken in one of seven high-income countries. In addition, only two studies identified through this work examined the perspectives of indigenous older adults specific to aged care design. More research is required, including in low and middle-income countries and with indigenous communities, where the ageing population is also burgeoning and cultural perspectives on aged care may influence both access to and design features required within RAC [64,65]. A better understanding of RAC-built environments in low-to-middle income regions, or with specific communities, can also inform design professionals and policymakers for evidence-based decision-making, whilst also building evidence of key considerations particular to global regions or communities [2].

The engagement of older adults in the design, use and evaluation of both built environment and assistive technology is viewed as integral to both good practice and the attainment of personally meaningful outcomes [4,8]. Whilst there have been recent calls for investment in participatory research focused on assistive technology and environment design that is undertaken with older adults with dementia [5], the current review found that a quarter (25%) of publications meeting the inclusion criteria did not include perspectives of aged care residents as part of the evaluation undertaken. Only four studies identified in this review used participatory methods (e.g., Photovoice) such that older adults of varying abilities could participate [44,66,67,68]. The current research demonstrates that more work is required to build an evidence base of built and technology design methods and measures that are informed by aged care services end users and can accommodate the range of abilities and support needs they may experience.

Measuring the impact of the RAC-built and technology environments on health and well-being can provide the evidence-based target interventions required in urban design and infrastructure development. However, the current review demonstrated variability in study design, participant type and number, interventions, outcomes, and measures used. More built environment and assistive technology design evaluation research is required, and—as other authors have also highlighted—close attention to the methodological design of prospective research will be important to both allow the building of an international body of evidence using aligned methods and measures and reduce risk of bias, improving certainty that it was, in fact, the environment that impacted outcomes identified [17].

This research has two main limitations that should be considered in the context of the results reported. Firstly, the two-staged process our team designed took significant time and human resources to complete. The scoping and reporting on key features of the literature identified were completed in full detail prior to the ICF mapping being undertaken. For this reason, there was a delay between publication retrieval and data extraction, and the mapping and reporting of the same. Following, specific to the second stage of work, mapping was undertaken of any work that contributed to the relevant ICF subdomain category, but did not weight the contribution by ICF subdomain. This meant that some publications may have offered an in-depth examination specific to a subdomain, whilst others only touched briefly on one. Finally, we acknowledge that this research has highlighted contemporary measures, methods and framing of design and technology, identifying a range of gaps. Whilst it is beyond the remit of this scoping review to explore whether these gaps are due to methodological challenges, disciplinary silos, funding priorities, technological limitations, or conceptual biases within the field, such investigations may be considered in future studies.

## 5. Conclusions

This review identified methods and measures used to evaluate RAC-built environments and assistive technology designs internationally, and examined the populations with whom these approaches were used. In addition, the review mapped the identified approaches to the WHO ICF. Together, this analysis provided a unique addition to the evidence base by providing researchers with both a taxonomy of tools and an analytic frame with which to understand their scope and limitations.

Analyzing the role of the built environment and assistive technology design in aged care can identify ways to facilitate or remove barriers to healthier spaces and lives for older adults; however, currently, the available body of research is limited. Further research is required to inform both policy and practice. Consideration of methods and measures that examine both the environment and activity and participation domains of the ICF should also be considered to holistically address the needs of older adults. The lack of attention to the full range of human participation domains within the ICF and the exclusion of the perspectives of service users/consumers in many studies is an identified issue. Taken together, these findings point to the imperative for future research to include participatory methods. Such methods should examine the experiences and perspectives of the multiple stakeholders who utilize these environments, particularly residents of aged care and their families.

## Figures and Tables

**Table 1:** Concepts and search terms.

Concept	Search Term
Target population: ≥18 years of age, or mixed populations with at least a 50% sub population that includes adults in residential aged care	“residential aged care” OR “homes for the aged” OR “Residential care” OR “Residential design” OR “Nursing Home” OR “long term care” OR “longterm care” OR “Aged care facilit*” OR “care home” OR “care facilit*” OR “Residential care” OR “institutionalised elders” OR “Institutionalised elderly” OR “institutionalized elder*” OR “skilled nursing facilit*” OR “Extended care adj2 facilit*” OR “geriatric adj2 (home* OR facilit* OR institution*)” OR “Long-term care adj2 (facilit* OR institution* OR setting* OR resident* OR provider*)” OR “LTC adj2 (facilit* OR institution* OR setting* OR resident* OR provider*)” OR “longterm care adj2 (facilit* OR institution* OR setting* OR resident* OR provider*)” OR “Residential adj2 (home* OR care OR facilit*)” OR “long-stay adj2 (facilit* or institution* or resident*)” AND
Topic of interest: Evaluation methods/measures	Survey OR Questionnaire OR Instrument OR Assessment OR Measur* OR Evaluat* OR Scale* OR Tool* OR “Self report” OR “Self-report” OR Inventor* OR Score OR Indicator OR Rating* OR Psychometric OR “reproducibility of results” OR Reliab* OR Valid* OR Reproducib* OR Standardis* OR Standardiz* OR Validat* AND
Context: Built and technology-enabled design	“Built design” OR “Design adj2 (build* OR technology OR Architect*))” OR “Technology design” OR “Technology support” OR “Technology-enabled” OR “Self-Help Devices” OR “Assistive Technology” OR “Smart technology” OR Architect*

**Table 2:** Descriptive information of methods used to collect data.

Method	Number of Articles	Percentage of Articles (n = 81)
Questionnaire/survey	39	48%
Observations	34	42%
Interviews	26	32%
Assessments	19	23%
Architectural drawings/designs	12	15%
Literature review	11	14%
Focus groups	10	12%
Space syntax/spatial layouts	10	5%
Behavior monitoring/mapping	4	5%
Environment monitoring	4	5%
Photographic documentation	4	5%
System/log sheets/diary	4	4%
Document analysis	3	4%
Design tool	3	2%
Field notes	2	2%
Delphi	2	1%
Workshops	1	1%
Audit of data/forms	1	1%
Concept mapping	1	1%

**Table 3:** (**a**)**.** Measures reported in studies, which were developed to evaluate built environment and/or assistive technology design in RAC. (**b**)**.** Published/standardized measures * used to evaluate built environment and/or assistive technology design in RAC.

**(a)**		
**Measure Developed**	**Brief Description**	
Corridor Coding System Scales (CCSS) [40]	Used to assess the quality/quantity of the indoor physical environment, including walking space dimensions (e.g., size, width, handrail, seating, destination, floor material, lighting, artwork, decoration, plants, window view, signage and obstruction.	
Environments for Ageing and Dementia Design Assessment Tool (EADDAT) [41]	Tool consisting of three tiers (dementia aware; dementia supportive; dementia inclusive) to help make homes, premises, and public places more accessible to an ageing population and those living with dementia.	
Global impression scale [42]	10-point scale to assess whether the outdoor spaces of a facility were likely to be encouraged to be used. The information is used to provide a single score from 1 (not likely to encourage use of outdoor space) to 10 (most likely to encourage use of outdoor space).	
Plan-EAT [43]	Plan-EAT is a floor-plan-based method designed to be used in the architectural design process. It provides a percentage-based overall score to assess the dementia design quality.	
PLANET Checklist [44]	Checklist that can be used to assess the potential for connection to nature (Person, Location, Architecture, Nature, Energy and Technology) for people living in dementia care environments.	
Sheffield Care Environment Assessment Matrix (SCEAM) tool [45]	Measures the physical environment on 11 user-related domains (privacy, personalization, choice and control, community, safety and health, support for physical frailty, comfort, support for cognitive frailty, awareness of outside world, normalness and authenticity, provision for staff).	
Satisfaction with Living Environment at Nursing Home Scale (SLE-NHS) [46]	18-item scale that measures residents’ satisfaction with their living environment and quality of life.	
User Needs Questionnaire [47]	Two-part questionnaire. Part A captures demographic information, daily activity and interest in new technologies. Part B collects information about the level of acceptance of proposed technical solutions, appearance, functionalities and interaction.	
(**b**)		
**Measures and Citation**	**Description**	**Citing Article(s)**
Dementia Services Development Centre (DSDC) audit tool [48]	76-item questionnaire structured into 13 sections used to rate the suitability of a building for people with dementia.	[49]
Environmental Audit Tool (EAT) [50]	Used to assess the quality of RAC environments for people with dementia. Consists of 72 questions across 10 sub-scales: safety; size; visual access; reduction in unnecessary stimuli; highlighting of useful stimuli; provision for wandering and outdoor area; familiarity; privacy and community; community links; and domestic activities.	[38,51,52,53]
Environmental Satisfaction [54]	Satisfaction with the living environment measured using a 5-point Likert scale (Strongly disagree to Strongly agree) with the item “I would rather live here than move to another home”.	[39]
Safe and Connected assessment tool [55]	Tool includes five architectural-design fields (Bubble concept; Outdoor space; Distancing space; Ventilation; Organization architectural measures) and assesses the quality and readiness of nursing homes to cope with situations such as the COVID-19 pandemic.	[56]
Semantic environmental description [57]	Standardized tool consisting of 36 adjectives rated on a scale from 1 (slightly) to 7 (very) that is intended for a participant to describe their perception of an interior, exterior or simulated environment across 8 dimensions (pleasantness; complexity; unity; enclosedness; potency; social status; affection; originality).	[58]
Swedish version of the Sheffield Care Environment Assessment Matrix (S-SCEAM) [59]	Adapted from the British version, to assess the environmental quality of Swedish RAC’s. Includes 210 items, each describing an environmental element across eight domains (cognitive support, physical support, safety, normalness, openness and integration, privacy, comfort, choice).	[60]
Therapeutic Environment Screening Survey of Nursing Homes (TESS-NH) [61]	Observational instrument used to assess the ability of physical environments to address therapeutic goals for persons with dementia. Contains 84 discrete items plus 1 global item that cover 13 domains (exit control, maintenance, cleanliness, safety, orientation/cueing, privacy, unit autonomy, outdoor access, lighting, noise, visual/tactile stimulation, space/seating, and familiarity/home likeness).	[51,62]

* defined as measurement instruments or tools with clear procedures for administration and scoring.

**Table 4:** Populations methods were used with.

	Population: n (%) of Articles							
Method	Older Adults (65+ Years)	Young People (<65 Years)	RAC Staff	Family Members	Architects/Planners	Experts in the Field of RAC	No Participants	Indigenous Populations
Questionnaire/survey	33 (41%)	7 (9%)	23 (28%)	5 (6%)	3 (4%)	1 (1%)	0 (0%)	1 (1%)
Observations	30 (37%)	4 (5%)	19 (23%)	7 (9%)	5 (6%)	0 (0%)	2 (2%)	0 (0%)
Interviews	20 (25%)	4 (5%)	22 (27%)	7 (9%)	6 (7%)	2 (2%)	0 (0%)	0 (0%)
Assessments	11 (14%)	1 (1%)	6 (7%)	0 (0%)	0 (0%)	0 (0%)	6 (7%)	1 (1%)
Architectural drawings/designs	8 (10%)	3 (4%)	6 (7%)	2 (2%)	2 (2%)	0 (0%)	3 (4%)	0 (0%)
Focus groups	5 (6%)	0 (0%)	10 (12%)	3 (5%)	2 (2%)	0 (0%)	0 (0%)	0 (0%)
Literature review	3 (4%)	1 (1%)	6 (7%)	2 (2%)	3 (4%)	2 (2%)	2 (2%)	0 (0%)
Behavior monitoring/mapping	4 (5%)	2 (2%)	4 (5%)	1 (1%)	3 (4%)	0 (0%)	0 (0%)	0 (0%)
Photographic documentation	4 (5%)	2 (2%)	2 (2%)	2 (2%)	0 (0%)	0 (0%)	0 (0%)	0 (0%)
System/log sheets/diary	4 (5%)	1 (1%)	4 (5%)	0 (0%)	0 (0%)	0 (0%)	0 (0%)	0 (0%)
Space syntax/ spatial layouts	3 (4%)	1 (1%)	0 (0%)	0 (0%)	0 (0%)	0 (0%)	7 (9%)	0 (0%)
Environment monitoring	2 (2%)	0 (0%)	3 (4%)	1 (1%)	0 (0%)	0 (0%)	1 (1%)	0 (0%)
Design tool	1 (1%)	0 (0%)	2 (2%)	1 (1%)	0 (0%)	0 (0%)	1 (1%)	0 (0%)
Field notes	2 (2%)	0 (0%)	1 (1%)	1 (1%)	0 (0%)	0 (0%)	0 (0%)	0 (0%)
Document analysis	1 (1%)	0 (0%)	2 (2%)	0 (0%)	0 (0%)	0 (0%)	1 (1%)	0 (0%)
Delphi	0 (0%)	0 (0%)	1 (1%)	0 (0%)	1 (1%)	2 (2%)	0 (0%)	0 (0%)
Concept mapping	1 (1%)	0 (0%)	1 (1%)	1 (1%)	0 (0%)	0 (0%)	0 (0%)	0 (0%)
Media coverage analysis	1 (1%)	0 (0%)	1 (1%)	0 (0%)	0 (0%)	0 (0%)	0 (0%)	0 (0%)
Workshops	0 (0%)	0 (0%)	1 (1%)	0 (0%)	0 (0%)	0 (0%)	0 (0%)	0 (0%)
Audit of data/forms	0 (0%)	0 (0%)	0 (0%)	0 (0%)	0 (0%)	0 (0%)	1 (1%)	0 (0%)
Total (any method)	52 (64%)	9 (11%)	41 (51%)	11 (14%)	7 (7%)	3 (4%)	16 (20%)	2 (2%)

**Table 5:** Populations measures were used with.

	Population: n (%) of Articles							
Measure	Older Adults (65+ Years)	Young People (<65 Years)	RAC Staff	Family Members	Architects/Planners	Experts in the Field of RAC	No Participants	Indigenous Populations
CCSS [40]	1	0	1	0	0	0	0	0
DSDC [48]	0	0	1	0	0	0	0	0
EAT [50]	2	1	0	0	0	0	2	1
EADDAT [41]	0	0	0	0	0	0	1	0
Environmental Satisfaction [54]	1	0	1	0	0	0	0	1
Global impression scale [42]	1	0	0	0	0	0	0	0
Plan-EAT [43]	0	0	0	0	0	0	1	0
PLANET Checklist [44]	1	0	1	1	0	0	0	0
Safe and Connected assessment tool [55]	1	0	0	0	0	0	0	0
Semantic environmental description [57]	1	0	1	0	0	0	0	0
SCEAM tool [45]	2	0	2	0	0	0	0	0
S-SCEAM [59]	0	0	0	0	0	0	1	0
SLE-NHS [46]	1	0	0	0	0	0	0	0
TESS-NH [61]	1	0	1	0	0	0	0	0
User Needs Questionnaire [47]	1	0	0	0	0	0	0	0

## Data Availability

Supporting data are available upon reasonable request made to the corresponding author.

## Supplementary Materials

The following supporting information can be downloaded at: https://www.mdpi.com/article/10.3390/ijerph23070869/s1, Table S1: Preferred Reporting Items for Systematic reviews and Meta-Analyses extension for Scoping Reviews (PRISMA-ScR) Checklist [37]; Table S2: Articles that met eligibility for inclusion in the review [28,38,39,40,41,42,43,44,45,46,47,49,51,52,53,55,56,58,60,62,66,67,68,69,70,71,72,73,74,75,76,77,78,79,80,81,82,83,84,85,86,87,88,89,90,91,92,93,94,95,96,97,98,99,100,101,102,103,104,105,106,107,108,109,110,111,112,113,114,115,116,117,118,119,120,121,122,123,124,125,126,127]; Table S3: Components of the ICF examined [28,38,39,40,41,42,43,44,45,46,47,49,51,52,53,55,56,58,60,62,66,67,68,69,70,71,72,73,74,75,76,77,78,79,80,81,82,83,84,85,86,87,88,89,90,91,92,93,94,95,96,97,98,99,100,101,102,103,104,105,106,107,108,109,110,111,112,113,114,115,116,117,118,119,120,121,122,123,124,125,126,127].

## Abbreviations

The following abbreviations are used in this manuscript:

CCSS	Corridor Coding System Scales
DOI	Digital Object Identifier
DSDC	Dementia Services Development Centre
EADDAT	Environments for Ageing and Dementia Design Assessment Tool
EAT	Environmental Audit Tool
ICF	International Classification of Functioning, Disability and Health
PRISMA-ScR	Preferred Reporting Items for Systematic Reviews and Meta-Analyses-extension for scoping reviews
RAC	Residential Aged Care
SLE-NHS	Satisfaction with Living Environment at Nursing Home Scale
SCEAM	Sheffield Care Environment Assessment Matrix tool
S-SCEAM	Swedish version of the Sheffield Care Environment Assessment Matrix
TESS-NH	Therapeutic Environment Screening Survey of Nursing Homes
WHO	World Health Organization
