# Supplementary material for: Built Environment and Assistive Technology Design in Residential Aged Care: A Scoping Review and Mapping of Evaluation Methods and Measures to the World Health Organization’s International Classification of Functioning, Disability and Health
Source: Int J Environ Res Public Health. 2026 Jul 3;23(7):869. doi: 10.3390/ijerph23070869 (PMC13410301; doi:10.3390/ijerph23070869)
Supplement: Supplementary file 1 [file ijerph-23-00869-s001.zip › ijerph-4159091-supplementary.pdf]

Table S1. Preferred Reporting Items for Systematic reviews and Meta-Analyses extension for Scoping Reviews (PRISMA-ScR) Checklist

SECTION	ITEM	PRISMA-ScR CHECKLIST ITEM	REPORTED ON PAGE #
**TITLE**			
Title	1	Identify the report as a scoping review.	1
**ABSTRACT**			
Structured summary	2	Provide a structured summary that includes (as applicable): background, objectives, eligibility criteria, sources of evidence, charting methods, results, and conclusions that relate to the review questions and objectives.	2
**INTRODUCTION**			
Rationale	3	Describe the rationale for the review in the context of what is already known. Explain why the review questions/objectives lend themselves to a scoping review approach.	2
Objectives	4	Provide an explicit statement of the questions and objectives being addressed with reference to their key elements (e.g., population or participants, concepts, and context) or other relevant key elements used to conceptualize the review questions and/or objectives.	2
**METHODS**			
Protocol and registration	5	Indicate whether a review protocol exists; state if and where it can be accessed (e.g., a Web address); and if available, provide registration information, including the registration number.	4
Eligibility criteria	6	Specify characteristics of the sources of evidence used as eligibility criteria (e.g., years considered, language, and publication status), and provide a rationale.	5
Information sources*	7	Describe all information sources in the search (e.g., databases with dates of coverage and contact with authors to identify additional sources), as well as the date the most recent search was executed.	5
Search	8	Present the full electronic search strategy for at least 1 database, including any limits used, such that it could be repeated.	5
Selection of sources of evidence†	9	State the process for selecting sources of evidence (i.e., screening and eligibility) included in the scoping review.	6
Data charting process‡	10	Describe the methods of charting data from the included sources of evidence (e.g., calibrated forms or forms that have been tested by the team before their use, and whether data charting was done independently or in duplicate) and any processes for obtaining and confirming data from investigators.	6
Data items	11	List and define all variables for which data were sought and any assumptions and simplifications made.	6
Critical appraisal of individual sources of evidence§	12	If done, provide a rationale for conducting a critical appraisal of included sources of evidence; describe the methods used and how this information was used in any data synthesis (if appropriate).	Not applicable

SECTION	ITEM	PRISMA-ScR CHECKLIST ITEM	REPORTED ON PAGE #
Synthesis of results	13	Describe the methods of handling and summarizing the data that were charted.	6
**RESULTS**			
Selection of sources of evidence	14	Give numbers of sources of evidence screened, assessed for eligibility, and included in the review, with reasons for exclusions at each stage, ideally using a flow diagram.	7
Characteristics of sources of evidence	15	For each source of evidence, present characteristics for which data were charted and provide the citations.	7-11
Critical appraisal within sources of evidence	16	If done, present data on critical appraisal of included sources of evidence (see item 12).	Not applicable
Results of individual sources of evidence	17	For each included source of evidence, present the relevant data that were charted that relate to the review questions and objectives.	7-11 and Tables S2 and S3
Synthesis of results	18	Summarize and/or present the charting results as they relate to the review questions and objectives.	7-11
**DISCUSSION**			
Summary of evidence	19	Summarize the main results (including an overview of concepts, themes, and types of evidence available), link to the review questions and objectives, and consider the relevance to key groups.	11-13
Limitations	20	Discuss the limitations of the scoping review process.	13
Conclusions	21	Provide a general interpretation of the results with respect to the review questions and objectives, as well as potential implications and/or next steps.	13
**FUNDING**			
Funding	22	Describe sources of funding for the included sources of evidence, as well as sources of funding for the scoping review. Describe the role of the funders of the scoping review.	14

JBI = Joanna Briggs Institute; PRISMA-ScR = Preferred Reporting Items for Systematic reviews and Meta-Analyses extension for Scoping Reviews.
* Where *sources of evidence* (see second footnote) are compiled from, such as bibliographic databases, social media platforms, and Web sites.
† A more inclusive/heterogeneous term used to account for the different types of evidence or data sources (e.g., quantitative and/or qualitative research, expert opinion, and policy documents) that may be eligible in a scoping review as opposed to only studies. This is not to be confused with *information sources* (see first footnote).
‡ The frameworks by Arksey and O'Malley (6) and Levac and colleagues (7) and the JBI guidance (4, 5) refer to the process of data extraction in a scoping review as data charting.
§ The process of systematically examining research evidence to assess its validity, results, and relevance before using it to inform a decision. This term is used for items 12 and 19 instead of "risk of bias" (which is more applicable to systematic reviews of interventions) to include and acknowledge the various sources of evidence that may be used in a scoping review (e.g., quantitative and/or qualitative research, expert opinion, and policy document).

From: Tricco AC, Lillie E, Zarin W, O'Brien KK, Colquhoun H, Levac D, et al. PRISMA Extension for Scoping Reviews (PRISMAScR): Checklist and Explanation. Ann Intern Med. 2018;169:467–473. doi: 10.7326/M18-0850.

Table S2. Articles that met eligibility for inclusion in the review.

Year	Author(s)	Article Title	DOI
2013	Aloulou, H.; Mokhtari, M.; Tiberghien, T.; Biswas, J.; Phua, C.; Kenneth Lin, J. H.; Yap, P.	Deployment of assistive living technology in a nursing home environment: methods and lessons learned	https://dx.doi.org/10.1186/1472-6947-13-42
2011	Andersson, J. E.	"Touching up" communal space of a residential home setting: A comparative study of tools for assessing changes in the interior architectural space	https://dx.doi.org/10.1080/02763893.2011.571085
2014	Andersson, M.; Ryd, N.; Malmqvist, I.	Exploring the function and use of common spaces in assisted living for older persons	https://dx.doi.org/10.1177/193758671400700308
2016	Beedholm, K.; Frederiksen, K.; Lomborg, K.	What Was (Also) at Stake When a Robot Bathtub Was Implemented in a Danish Elder Center: A Constructivist Secondary Qualitative Analysis	https://dx.doi.org/10.1177/1049732315586550
2015	Bengtsson, A.; Hägerhäll, C.; Englund, J. E.; Grahn, P.	Outdoor Environments at Three Nursing Homes: Semantic Environmental Descriptions	https://dx.doi.org/10.1177/1049732315586550
2015	Brodersen, S.; Lindegaard, H.	The smart floor: How a public-private partnership codeveloped a heterogeneous healthcare technology system	https://dx.doi.org/10.3233/978-1-61499-560-9-191
2023	Cao, W.; Dewancker, B.	Interpreting spatial layouts of nursing homes based on partitioning theory	https://dx.doi.org/10.1080/13467581.2021.2007110
2021	Carnemolla, P.; Debono, D.; Hourihan, F.; Hor, S.; Robertson, H.; Travaglia, J.	The influence of the built environment in enacting a household model of residential aged care for people living with a mental health condition: A qualitative post-occupancy evaluation	https://dx.doi.org/10.1016/j.healthplace.2021.102624
2020	Carta, S.; Loe, S. S.; Turchi, T.; Simon, J.	Self-organising floor plans in care homes	https://dx.doi.org/10.3390/su12114393
2006	Celler, B. G.; Basilakis, J.; Budge, M.; Lovel, N. H.	A clinical monitoring and management system for residential aged care facilities	

2007	Chalfont, G. E.	Wholistic design in dementia care: Connection to nature with PLANET	https://dx.doi.org/10.1300/J081v21n01_08
2013	Chang, C. H.; Lu, M. S.; Lin, T. E.; Chen, C. H.	The effectiveness of visual art on environment in nursing home	https://dx.doi.org/10.1111/jnu.12011
2018	Chau, H.; Newton, Clare; W., Catherine M. M.; Ma, N.; Wang, J.	Design lessons from three Australian dementia support facilities	
2008	Chu, M.T.; Khosla, R.; Khaksar, S. M. S.; Nguyen, K.	Service innovation through social robot engagement to improve dementia care quality	https://dx.doi.org/10.1080/10400435.2016.1171807
2009	Allen, D.	Designed for a better life	https://dx.doi.org/10.7748/nop.21.3.28.s31
2007	Detweiler, M. B.; Murphy, P. F.; Myers, L. C.; Kim, K. Y.	Does a wander garden influence inappropriate behaviors in dementia residents?	https://dx.doi.org/10.1177/1533317507309799
2015	Durvasula, S.; Mason, R. S.; Kok, C.; Macara, M.; Parmenter, T. R.; Cameron, I. D.	Outdoor areas of Australian residential aged care facilities do not facilitate appropriate sun exposure	https://dx.doi.org/10.1071/AH14035
2021	Edwards, K. J.; Jones, R. B.; Shenton, D.; Page, T.; Maramba, I.; Warren, A.; Fraser, F.; Križaj, T.; Coombe, T.; Cowls, H.; Chatterjee, A.	The Use of Smart Speakers in Care Home Residents: Implementation Study	https://dx.doi.org/10.2196/26767
2015	Ferdous, F.; Moore, K. D.	Field observations into the environmental soul: Spatial configuration and social life for people experiencing dementia	https://dx.doi.org/10.1177/1533317514545378
2011	Fleming, R.	An environmental audit tool suitable for use in homelike facilities for people with dementia	https://dx.doi.org/10.1111/j.1741-6612.2010.00444.x
2015	Fleming, R.; Bennett, K.	Assessing the quality of environmental design of nursing homes for people with dementia: Development of a new tool	https://dx.doi.org/10.1111/ajag.12233
2014	Fleming, R.; Goodenough, B.; Low, L. F.; Chenoweth, L.; Brodaty, H.	The relationship between the quality of the built environment and the quality of life of people with dementia in residential care	https://dx.doi.org/10.1177/1471301214532460
2021	Francis, K.; Murtha, S.	The inclusion and efficacy of first-person narrative in the design of long-term care homes	https://dx.doi.org/10.1080/00038628.2021.1917336
2021	Franke, A.; Nass, E.; Piereth, A. K.; Zettl, A.; Heidl, C.	Implementation of Assistive Technologies and Robotics in Long-Term Care Facilities: A Three-Stage Assessment Based on Acceptance, Ethics, and Emotions	https://dx.doi.org/10.3389/fpsyg.2021.694297
2010	Hadjri, K.; Faith, V.; McManus, M.	Designing dementia nursing and residential care homes	https://dx.doi.org/10.1108/14769011211270765

2017	Hegde, A. L.	Environmental lighting in nursing homes: A comparison of agency standards that regulate nursing homes with industry ANSI/IES RP-28 lighting standards	https://dx.doi.org/10.18848/2325-1328/CGP/v12i01/1-16
2007	Hernandez, R. O.	Effects of therapeutic gardens in special care units for people<br>with dementia: Two case studies	https://dx.doi.org/10.1300/J081v21n01_07
2014	Hsieh, Y.-P.	Users' perceptions of bedroom privacy and personalization<br>in long-term care facilities	https://dx.doi.org/10.3130/jaabe.13.625
2022	Hung, L.; Mann, J.; Perry, J.; Berndt, A.; Wong, J.	Technological risks and ethical implications of using robots<br>in long-term care	https://dx.doi.org/10.1177/20556683221106917
2017	Khaksar, S. M. S.; Shahmehr, F. S.; Khosla, R.; Chu, M. T.	Dynamic capabilities in aged care service innovation: the role<br>of social assistive technologies and consumer-directed<br>care strategy	https://dx.doi.org/10.1108/JSM-06-2016-0243
2021	Khaksar, S. M. S.; Khosla, R.; Singaraju, S.; Slade, B.	Carer’s perception on social assistive technology acceptance<br>and adoption: moderating effects of perceived risks	https://dx.doi.org/10.1080/0144929X.2019.1690046
2017	Khosla, R.; Nguyen, K.; Chu, M. T.	Human Robot Engagement and Acceptability in	https://dx.doi.org/10.1080/10447318.

		Residential<br>Aged Care	2016.1275435
2016	Koceski, S.; Koceska, N.	Evaluation of an Assistive Telepresence Robot for Elderly Healthcare	https://dx.doi.org/10.1007/s10916-016-0481-x
2019	Kriegel, J.; Grabner, V.; Tuttle-Weidinger, L.; Ehrenmuller, I.	Socially Assistive Robots (SAR) in In-Patient Care for the Elderly	https://dx.doi.org/10.3233/978-1-61499-971-3-178
2017	Kymäläinen, T.; Plomp, J.; Tuomisto, T.; HeinilÃ¤, J.; Urhemaa, T.	Designing smart living for ageing Alice - and the persons next door	https://dx.doi.org/10.1080/17508975.2015.1005566
2019	Landi, D.	'The Open Typology': Towards Socially Sustainable Architectural and Care Types	https://dx.doi.org/10.14324/111.444.amps.2019v16i1.001
2020	Landi, D.; Smith, G.	The implications of a new paradigm of care on the built environment. The Humanitas© Deventer model: Innovative practice	https://dx.doi.org/10.1177/1471301219845480
2020	Lee, J.; Lee, H.	Employing visibility and agent-based accessibility analysis to enhance social interactions in older adult care facilities	https://doi.org/10.1080/00038628.2020.1719819
2017	Lee, J. H.; Ostwald, M. J.; Lee, H.	Measuring the spatial and social characteristics of the architectural plans of aged care facilities	https://dx.doi.org/10.1016/j.foar.2017.09.003
2015	Lu, Z.; Rodiek, S.; Shepley, M. M.; Tassinary, L. G.	Environmental influences on indoor walking behaviours of assisted living residents	https://dx.doi.org/10.1080/09613218.2015.1049494
2018	Martens, I.; Verbeek, H.; Aarts, J.; Bosems, W. P. H.; Felix, E.; van Hoof, J.	The vision of bedfast nursing home residents of their quality of life and the contribution of technological innovations in and around the bed	https://dx.doi.org/10.1108/JET-01-2018-0003
2020	McGann, S.; Bulsara, C.; Farley, H.	Socio-spatial and quality of life themes in aged care	https://dx.doi.org/10.1111/jan.14497

		architecture: A qualitative methods protocol	
2020	Melkas, H.; Hennala, L.; Pekkarinen, S.; Kyrki, V.	Impacts of robot implementation on care personnel and clients in elderly-care institutions	https://dx.doi.org/10.1016/j.ijmedinf.2019.104041
2021	Mu, J.; Kang, J.; Wu, Y.	Acoustic environment of comprehensive activity spaces in nursing homes: A case study in Harbin, China	https://dx.doi.org/10.1016/j.apacoust.2021.107932
2022	Mu, J.; Zhang, S.; Kang, J.	Estimation of the quality of life in housing for the elderly based on a structural equation model	https://dx.doi.org/10.1007/s10901-021-09887-0
2018	Noguchi, M.; Ma, N.; Woo, C. M. M.; Chau, H. W.; Zhou, J.	The usability study of a proposed environmental experience design framework for active ageing	https://dx.doi.org/10.3390/buildings8120167
2017	Nordin, S.; McKee, K.; Wijk, H.; Elf, M.	Exploring Environmental Variation in Residential Care Facilities for Older People	https://dx.doi.org/10.1177/1937586716648703
2021	Oatley, G.; Choudhury, T.; Buckman, P.	Smart textiles for improved quality of life and cognitive assessment	https://dx.doi.org/10.3390/s21238008
2020	Obayashi, K.; Kodate, N.; Masuyama, S.	Measuring the impact of age, gender and dementia on communication-robot interventions in residential care homes	https://dx.doi.org/10.1111/ggi.13890
2020	Obayashi, K.; Kodate, N.; Masuyama, S.	Can connected technologies improve sleep quality and safety of older adults and care-givers? An evaluation study of sleep monitors and communicative robots at a residential care home in Japan	https://dx.doi.org/10.1016/j.techsoc.2020.101318
2022	Obayashi, K.; Kodate, N.; Masuyama, S.	Assessing the Impact of an Original Soft Communicative Robot in a Nursing Home in Japan: Will Softness or Conversations Bring more Smiles to Older People?	https://dx.doi.org/10.1007/s12369-021-00815-4

2012	Paiva, M. M. B.; Villarouco, V.	Accessibility in collective housing for the elderly: a case study in Portugal	https://dx.doi.org/10.3233/wor-2012-0716-4174
2004	Parker, C.; Barnes, S.; McKee, K.; Morgan, K.; Torrington, J.; Tregenza, P.	Quality of life and building design in residential and nursing homes for older people	https://dx.doi.org/10.1017/S0144686X04002387
2018	Peng, J.; Zeng, Y.; Zhao, L.; Zeng, J.	An investigation of acoustical environments in the elderly care facilities	https://dx.doi.org/10.1016/j.apacoust.2018.03.009
2023	Quirke, M.; Ostwald, M.; Fleming, R.; Taylor, M.; Williams, A.	A design assessment tool for layout planning in residential care for dementia	https://dx.doi.org/10.1080/00038628.2021.1984869
2022	Rom, Y.; Palgi, Y.; Isaacson, M.	Analyzing the Layout of Long-Term Care Facilities: A Psycho-Spatial Approach	https://dx.doi.org/10.1177/19375867211064538
2010	Schikhof, Y.; Mulder, I.; Choenni, S.	Who will watch (over) me? Humane monitoring in dementia care	https://dx.doi.org/10.1016/j.ijhcs.2010.02.002
2014	Scott, T. L.; Masser, B. M.; Pachana, N. A.	Multisensory installations in residential aged-care facilities: Increasing novelty and encouraging social engagement through modest environmental changes	https://dx.doi.org/10.3928/00989134-20140731-01
2017	Sima, L.	Are private bedrooms necessary for residential facilities for the elderly in China? - A study on residents' preferences in Shanghai	https://dx.doi.org/10.3130/aijt.23.219
2023	Klemenčič & Leskovar	The role of open space and green areas in the design of an inclusive nursing home environment during Covid-19 period	https://dx.doi.org/10.1016/j.egyr.2022.11.155
2012	Smith, R.; Fleming, R.; Chenoweth, L.; Jeon, Y. H.; Stein-Parbury, J.; Brodaty, H.	Validation of the Environmental Audit Tool in both purpose-built and non-purpose-built dementia care settings	https://dx.doi.org/10.1111/j.1741-6612.2011.00559.x

2023	Spinsante, S.; Poli, A.; Mongay Batalla, J.; et al	Clinically-validated technologies for assisted living: The vINCI project	https://dx.doi.org/10.1007/s12652-021-03419-y
2018	Tao, Y.; Gou, Z.; Lau, S. S. Y.; Lu, Y.; Fu, J.	Legibility of floor plans and wayfinding satisfaction of residents in Care and Attention homes in Hong Kong	https://dx.doi.org/10.1111/ajag.12574
2018	Tao, Y.; Lau, S. S. Y.; Gou, Z.; Fu, J.; Jiang, B.; Chen, X.	Privacy and Well-Being in Aged Care Facilities with a Crowded Living Environment: Case Study of Hong Kong Care and Attention Homes	https://dx.doi.org/10.3390/ijerph15102157
2020	Thomas, P.; Aletta, F.; Filipan, K.; Mynsbrugge, T. V.; De Geetere, L.; Dijckmans, A.; Botteldooren, D.; Petrovic, M.; Van de Velde, D.; De Vriendt, P.; Devos, P.	Noise environments in nursing homes: An overview of the literature and a case study in Flanders with quantitative and qualitative methods	https://dx.doi.org/10.1016/j.apacoust.2019.107103
2004	Torrington, J.; Barnes, S.; McKee, K.; Morgan, K.; Tregenza, P.	The influence of building design on the quality of life of older people	https://dx.doi.org/10.1080/00038628.2004.9697043
2020	Tuaycharoen, N.	Lighting to enhance wayfinding for Thai elderly adults in nursing homes	https://dx.doi.org/10.15627/jd.2020.3
2011	Valkila, N.; Saari, A.	The productivity impact of the voice link between elderly and nurses: An assisted living facility pilot	https://dx.doi.org/10.1016/j.archger.2010.05.008
2019	Van Hecke, L.; Van Steenwinkel, I.; Heylighen, A.	How Enclosure and Spatial Organization Affect Residents' Use and Experience of a Dementia Special Care Unit: A Case Study	https://dx.doi.org/10.1177/1937586718796614
2017	Van Steenwinkel, I.; Dierckx de Casterle, B.; Heylighen, A.	How architectural design affords experiences of freedom in residential care for older people	https://dx.doi.org/10.1016/j.jaging.2017.05.001
2021	Varshawsky, A. L.; Traynor, V.	Graphic designed bedroom doors to support dementia wandering in residential care homes: Innovative practice	https://dx.doi.org/10.1177/1471301219868619

2006	Wang, C. H.; Kuo, N. W.	Zeitgeists and development trends in long-term care facility design	
2018	Werner, C.; Moustris, G. P.; Tzafestas, C. S.; Hauer, K.	User-Oriented Evaluation of a Robotic Rollator That Provides Navigation Assistance in Frail Older Adults with and without Cognitive Impairment	https://dx.doi.org/10.1159/000484663
2010	Wigg, J. M.	Liberating the wanderers: Using technology to unlock doors for those living with dementia	https://dx.doi.org/10.1111/j.1467-9566.2009.01221.x
2014	Wong, J. K.; Skitmore, M.; Buys, L.; Wang, K.	The effects of the indoor environment of residential care homes on dementia suffers in Hong Kong: A critical incident technique approach	https://dx.doi.org/10.1016/j.buildenv.2013.12.001
2019	Woo, C. M. M.; Chau, H.; Zhou, J.; Pianella, A.; Newton, C.; Noguchi, M.	Physical and perceptual gap in indoor environmental quality : a mixed method study of space and users at an aged care facility in Victoria	
2020	Xie, H.; Zhong, B.; Liu, C.	Sound environment quality in nursing units in Chinese nursing homes: A pilot study	https://dx.doi.org/10.1177/1351010X20914237
2017	Yu, J.; Ma, G.; Chen, Y.	Comparison of the Built Environment of Homes for the Aged in China by Postoccupancy Evaluation	https://dx.doi.org/10.1061/(ASCE)AE.1943-5568.0000264
2017	Yu, J.; Ma, G.; Jiang, X.	Impact of the built environment and care services within rural nursing homes in China on quality of life for elderly residents	https://dx.doi.org/10.1108/ECAM-08-2016-0187
2020	Zarghami, E.; Olfat, M.; Haverinen-Shaughnessy, U.; Fatourehchi, D.	Development of an education integrated design framework for the physical space of nursing homes in relation to life expectancy of the older people	https://dx.doi.org/10.1111/hsc.12884
2022	Zhu, X.; Lee, H.; Sang, H.; Muller, J.; Yang, H.; Lee, C.; Ory, M.	Nursing Home Design and COVID-19: Implications for Guidelines and Regulation	https://dx.doi.org/10.1016/j.jamda.2021.12.026

Table S3. Components of the ICF examined.

	ICF – Environmental factors						ICF - Activities and Participation								
First author (year)	Products & technology	Built environ-ment (indoor)	Built environ-ment (outdoor)	Natural environ-ment	Support and relationships and attitudes	Services, systems and policies	Self-care	Interpersonal interactions and relationships	Communication	Learning & applying knowledge	Domestic life	Community, social, civic life	Mobility	Major life areas (education, work)
Aloulou, et al (2013)	•						•	•						
Andersson (2011)	•	•						•					•	
Andersson et al. (2014)		•			•	•	•		•					
Beedholm et al. (2016)	•				•		•		•					
Bengtsson et al. (2015)			•									•		
Brodersen et al. (2015)	•						•		•					
Cao et al. (2023)		•				•							•	
Carnemolla et al. (2023)		•			•		•	•				•		
Carta et al. (2020)	•	•		•		•	•							
Celler et al. (2006)	•					•								
Chalfont	•	•	•	•							•		•	

St. Michael's
Inspired Care.
Inspiring Science.

First author (year)	ICF – Environmental factors						ICF - Activities and Participation							
	Products & technology	Built environ-ment (indoor)	Built environ-ment (outdoor)	Natural environ-ment	Support and relationships and attitudes	Services, systems and policies	Self-care	Interpersonal interactions and relationships	Communication	Learning & applying knowledge	Domestic life	Community, social, civic life	Mobility	Major life areas (education, work)
(2007)														
Chang et al. (2013)		•										•		
Chau et al. (2018)		•	•					•			•			
Chu et al. (2017)	•					•		•						
Cunningham (2009)		•	•		•	•					•			
Detweiler et al. (2008)			•	•		•	•							
Durvasula et al. (2015)			•	•		•	•	•				•		
Edwards et al. (2021)	•					•		•		•	•			
Ferdous et al. (2015)		•				•		•						
Fleming (2011)		•	•								•			
Fleming et al. (2015)		•						•			•	•		
Fleming et al. (2016)		•	•			•					•	•		
Francis et al.		•	•	•			•	•	•			•		

	ICF – Environmental factors						ICF - Activities and Participation							
First author (year)	Products & technology	Built environ-ment (indoor)	Built environ-ment (outdoor)	Natural environ-ment	Support and relationships and attitudes	Services, systems and policies	Self-care	Interpersonal interactions and relationships	Communication	Learning & applying knowledge	Domestic life	Community, social, civic life	Mobility	Major life areas (education, work)
(2021)														
Franke et al. (2021)	•				•	•		•	•				•	
Hadjri et al. (2012)		•				•					•			
Hegde (2017)				•		•						•	•	
Hernandez (2007)			•	•				•			•	•	•	
Hsieh (2014)		•						•	•			•		
Hung et al. (2022)	•				•	•		•				•	•	
Khaksar et al. (2017)	•				•	•		•			•	•		
Khaksar et al. (2021)	•				•	•			•					
Khosla et al. (2017)	•							•				•		
Klemenčič et al. (2023)			•					•			•			
Kocesk et al. (2016)	•						•	•			•			
Kriegel et al. (2019)	•					•		•						

First author (year)	ICF – Environmental factors						ICF - Activities and Participation							
	Products & technology	Built environ-ment (indoor)	Built environ-ment (outdoor)	Natural environ-ment	Support and relationships and attitudes	Services, systems and policies	Self-care	Interpersonal interactions and relationships	Communication	Learning & applying knowledge	Domestic life	Community, social, civic life	Mobility	Major life areas (education, work)
Kymäläinen et al. (2017)	•			•		•								
Landi (2019)		•	•		•	•		•				•		
Landi et al. (2020)	•	•	•		•	•		•	•	•		•		
Lee et al. (2020)	•	•			•			•					•	
Lee at el. (2017)		•						•						
Lu et al. (2015)		•	•			•						•	•	
Martens et al. (2018)	•				•			•			•	•	•	
McGann et al. (2020)		•				•		•			•			
Melkas et al. (2020)	•				•	•		•						•
Mu et al. (2021)		•		•	•	•					•	•		
Mu et al. (2022)		•		•	•	•					•	•		
Noguchi et al. (2018)		•	•	•		•						•	•	
Nordin et al.		•	•			•		•			•			

First author (year)	ICF – Environmental factors: Products & technology	Built environ-ment (indoor)	Built environ-ment (outdoor)	Natural environ-ment	Support and relationships and attitudes	Services, systems and policies	ICF - Activities and Participation: Self-care	Interpersonal interactions and relationships	Communication	Learning & applying knowledge	Domestic life	Community, social, civic life	Mobility	Major life areas (education, work)
(2017)														
Oatley et al. (2021)	•										•			
Obayashi et al. (2020a)	•				•	•	•	•	•			•		
Obayashi et al. (2020b)	•					•	•				•			
Obayashi et al. (2022)	•				•	•			•			•		
Paiva et al. (2012)		•				•	•				•	•	•	
Parker et al. (2004)		•	•		•	•					•	•	•	
Peng et al. (2018)				•					•			-		
Quirke et al. (2023)		•	•								•	•	•	
Rom et al. (2022)		•		•		•					•			•
Schikhof et al. (2010)	•				•	•					•			
Sima (2017)		•		•	•	•		•			•			
Smith et al.		•		•	•	•					•	•		•

	ICF – Environmental factors						ICF - Activities and Participation								
First author (year)	Products & technology	Built environ-ment (indoor)	Built environ-ment (outdoor)	Natural environ-ment	Support and relationships and attitudes	Services, systems and policies	Self-care	Interpersonal interactions and relationships	Communication	Learning & applying knowledge	Domestic life	Community, social, civic life	Mobility	Major life areas (education, work)
(2012)														
Spinsante et al. (2023)		•				•					•	•		
Tao et al. (2018a)	•					•		•	•		•	•		
Tao et al. (2018b)		•				•						•		
Thomas et al. (2020)		•				•		•	•		•			
Torrington et al. (2004)		•		•	•	•		•				•		
Tuaycharoen (2020)	•	•	•			•						•	•	
Valkila et al. (2011)		•	•	•		•						•		
Van Hecke et al. (2019)	•				•				•		•			•
Van Steenwinkel et al. (2017)		•	•			•		•				•	•	
Varshawsky et al. (2021)		•				•		•				•		
Wang et al.		•				•					•	•	•	

First author (year)	ICF – Environmental factors						ICF - Activities and Participation							
	Products & technology	Built environ-ment (indoor)	Built environ-ment (outdoor)	Natural environ-ment	Support and relationships and attitudes	Services, systems and policies	Self-care	Interpersonal interactions and relationships	Communication	Learning & applying knowledge	Domestic life	Community, social, civic life	Mobility	Major life areas (education, work)
(2006)														
Werner et al. (2018)		•				•		•				•		
Wigg (2010)	•							•					•	
Wong et al. (2014)	•		•		•			•			•	•	•	
Woo et al. (2019)		•		•		•					•			
Xie et al. (2020)		•		•							•	•		
Yu et al. (2017a)		•		•		•	•				•			•
Yu et al. (2017b)		•	•	•		•		•			•	•	•	
Zarghami et al. (2020)		•	•	•		•		•				•	•	
Zhu et al. (2022)	•	•					•	•		•		•		

• = examined in the publication
